# Building Capacity of Community Nurses to Strengthen the Management of Uncomplicated Hypertension in Persons Living with HIV in Low- and Middle-Income Countries

**DOI:** 10.5334/gh.1216

**Published:** 2023-07-11

**Authors:** Dike Ojji, Angela Aifah, Juliet Iwelunmor, Erinn M. Hade, Deborah Onakomaiya, Calvin Colvin, Shivani Mishra, Nafesa Kanneh, Ashlin Rakhra, Gabriel Shedul, Daniel Henry, Adrian Duah, Daphne Lew, Geetha P. Bansal, Angela Attah, Gbenga Ogedegbe, Anyiekere Ekanem

**Affiliations:** 1 Department of Internal Medicine, Faculty of Clinical Sciences, College of Health Sciences, University of Abuja, Gwagwalada, Abuja, Nigeria; 2 Cardiovascular Research Unit, University of Abuja, and University of Abuja Teaching Hospital, Gwagwalada, Abuja, Nigeria; 3 Institute for Excellence in Health Equity (IEHE), New York University Grossman School of Medicine, New York, USA; 4 Department of Behavioral Science and Health Education, College for Public Health and Social Justice Saint Louis University, USA; 5 Department of Population Health, New York University Grossman School of Medicine, New York, USA; 6 Vilcek Institute of Graduate Biomedical Sciences, New York University Grossman School of Medicine, New York, USA; 7 Department of Family Medicine, University of Abuja Teaching Hospital, Gwagwalada, Abuja, Nigeria; 8 Institute for Excellence in Health Equity, New York University Grossman School of Medicine, New York, USA; 9 Division of Biostatistics, Washington University in St. Louis School of Medicine, St. Louis, USA; 10 Fogarty International Center, NIH, USA; 11 Akwa Ibom Primary Healthcare Development Agency, State Primary Health Care Development Board, Akwa Ibom State, Nigeria; 12 Department of Community Medicine, Faculty of Clinical Sciences, University of Uyo, Akwa Ibom State, Nigeria

**Keywords:** Capacity Building, Community Nurses, Hypertension Management, HIV Patients

## Abstract

**Objectives:**

Poor training of non-physician healthcare workers (especially community nurses) could hinder the successful integration of cardiovascular disease (CVD) management into HIV chronic care in primary healthcare facilities in low- and middle-income countries. To address this limitation, we included a holistic training programme with a robust module for both practice facilitators and community nurses as part of the formative stages of the managing hypertension among people living with HIV: an integrated model (MAP-IT), which is a study that is evaluating the effectiveness of practice facilitation on the integration of a task-strengthening strategy for hypertension control (TASSH) into primary healthcare centres in Akwa Ibom State of Nigeria.

**Methods:**

Between June and November 2021, 3 didactic training workshops were conducted using a training module which is based on the simplified Nigerian Hypertension Protocol for primary care and the World Health Organization (WHO) heart package. Knowledge acquired by the participants was assessed using anonymized pre- and post-training assessments in the first two workshops. Participants’ view of the training was assessed using a comprehensive course evaluation questionnaire.

**Results:**

A total of 92 community nurses and six practice facilitators were trained in the workshops on managing hypertension in persons living with HIV. Mean pre- and post-test scores improved from 11.9(3.4) to 15.9(2.9); p < 0.001 in the first workshop, and from 15.4(0.9) to 16.4 (1.4); p < 0.001 in the second workshop. The methodology used in the training, understanding of the MAP-IT study programme, and the level of engagement was highly rated by the participants with LIKERT scores of 3.2/4.0, 3.2/4.0, and 3.1/4.0 respectively.

**Conclusion:**

Our training methodology, which involved the train-the-trainer model to deliver simplified HIV and HTN care guidelines, showed improvement in the knowledge of managing hypertension in persons living with HIV and was highly rated by participants.

## Background

Despite the availability of evidence-based interventions for cardiovascular disease (CVD) control, including the control of hypertension, the healthcare workforce shortage in Africa limits the effective integration of these interventions into existing services, particularly to address the increasing prevalence of non-communicable diseases among people living with HIV [1,2,3]. For example, in Nigeria there were 3.8 physicians and 11.8 nurses per 10,000 populations in 2018 [4]. With the rapid emigration of healthcare workers from Nigeria to the United Kingdom, North America, and the Middle East, these estimates are likely lower than this [5]. Such an acute shortage of physicians, limits Nigeria’s capacity to offer hypertension services in primary health centers (PHCs), where the majority of persons living with HIV (PLWH) receive their care. With primary health care remaining the central approach of Nigeria’s healthcare delivery policy since 1988 targeted efforts to address the shortage of the healthcare workforce in PHCs will be critical to effectively integrate CVD management into the HIV care case [6,7].

In addition to the shortage in healthcare workforce, evidence shows that in low- and middle-income countries (LMICs) health professionals lack the requisite knowledge and skills to treat NCDs like hypertension due in part to poor training in diagnosis and management [6,7,8]. Studies in Nigeria highlight that the gap in knowledge and awareness among healthcare providers, particularly non-physician workers, remains a barrier to treating hypertension and other NCDs in PHCs [6]. These findings are of particular note as evidence suggests that lifestyle counseling provided by nurses is known to be effective in modifying patients’ health behaviors [6,9]. Furthermore, the emphasis on health education and training of the healthcare workforce, including non-physician workers, will be essential in addressing the shortage of physicians, the increasing prevalence of NCDs among the general population, and targeting populations already burdened with chronic conditions such as PLWH [6,7,10,11].

To mitigate the healthcare workforce shortage, the Nigerian Federal Ministry of Health (FMOH) enacted a task-shifting and task-sharing policy where non-physician healthcare workers are mandated to carry out clinical duties at PHCs across its healthcare system [6]. The initial policy was limited to case identification, patient counseling, treatment for maternal and child health, tuberculosis, malaria, and HIV, however, there has been a recent expansion of this policy to include NCDs, especially CVDs, and their risk factors [6,12]. Benefits of this strategy include increased access to treatment and subsequent health of individuals, improvement in the skills of the non-physician healthcare workers and greater efficiency of the health system [10]. Although the “Task-shifting and Task-sharing Policy for Essential Health Care Services in Nigeria” for hypertension is now being piloted in some PHCs, it has not been leveraged as a strategy to integrate NCD management into HIV care within PHCs, particularly in Akwa-Ibom State which has the highest burden of HIV prevalence in Nigeria [9].

To bridge this gap, we are conducting the “Managing Hypertension Among People Living with HIV: an InTegrated Model (MAP-IT)” study to evaluate the effectiveness of practice facilitation on the integration of a task-strengthening strategy for hypertension control (TASSH) into PHCs. Practice facilitation is a healthcare delivery strategy which focuses on the implementation and adoption of evidence-based care within primary care practices through supportive service provided by trained or skilled individuals [13]. TASSH is an evidence-based hypertension management training programme which leverages the WHO package of essential noncommunicable (PEN) disease interventions for primary health care to train nurses using ICTR – identifying patients with hypertension, providing lifestyle counseling, treating these patients using antihypertensive medication based on standard treatment and drug titration protocol, and referring complicated hypertension cases to physicians [14].

While our MAP-IT study is novel and has a robust study design, a critical health system challenge in Nigeria that could hinder the success of our programme is the lack of training of non-physician healthcare workers (community nurses included) to address NCDs, especially CVD at primary health facilities. To address this challenge, we included an evidence-based, holistic training programme with modules for both practice facilitators (PFs) and community nurses (CNs) as part of the formative stages of the MAP-IT study. The training module is based on the simplified Nigerian Hypertension Protocol for primary care, and the World Health Organization (WHO) hearts package, was initially created by the TASSH study in Ghana which included investigators from our study and was subsequently adapted by MAP-IT investigators and staff [8,15,16]. This manuscript describes our experience training both PFs and CNs in the formative stages of our work. The training manual is attached as a supplementary material.

## Methods

### The MAP-IT Study

Based in Akwa Ibom State, the MAP-IT study is being implemented in 30 PHCs, uses a stepped wedge cluster randomized trial (CRT) study design, and is guided by two implementation science frameworks, the integrated Promoting Action on Research Implementation in Health Services (i-PARiHS), and the Reach, Effectiveness, Adoption, Implementation, and Maintenance (RE-AIM) [17,18]. The study trains CNs working in PHCs that provide HIV services to deliver the TASSH programme for PLWH with uncontrolled hypertension. As previously noted, the TASSH programme is comprised of the Identify, Counsel, Treat, and Refer (ICTR) approach for controlling hypertension. CN are trained on these components and taught to deliver TASSH using the ‘5A’s’: Ask, Assess, Advise, Assist, and Arrange. A well-known behavior change model, the 5As has been used in primary care settings for several health conditions including smoking cessation [15]. For their part in the MAP-IT study, PFs are also trained on the TASSH programme and to provide support CNs as they deliver TASSH in PHCs. Below, we provide details on the training modules for both CNs and PFs.

### Training Modules

Three didactic training workshops were conducted in June 2021, July 2021, and November 2021. Each training comprised 1–2 days of general training for both PFs and CNs. PFs here are retired senior nurses who have a good knowledge of the primary healthcare system having worked in that system for 30 years and above.

An extra day’s training for the PFs on engaging, enhancing, and evaluating the CNs was conducted during the July 2021 training. The 5As are mapped onto the ICTR approach for managing PWLH with uncontrolled hypertension. The CNs were trained to administer TASSH to eligible patients by utilizing the 5As, based on the ICTR approach which involves the identification of patients with hypertension, counselling those with hypertension, treating those with mild to moderate (blood pressure of 140/90 mmHg to 179/109mmHg) hypertension, and referring those with severely elevated blood pressure (180/110mmHg and above) or with complicated hypertension (comorbid kidney disease, diabetes mellitus, heart attack, stroke, and heart failure). The modules were delivered to both CNs and PFs using didactic lectures, interactive workshops, role plays, and practical demonstrations.

- *Ask*: CNs were trained to *identify* elevated blood pressure by using a semi-automated blood pressure measurement device to screen PLWH who are 18 years of age or older and who come into the clinic. CNs were also trained to *ask* a series of relevant questions to exclude the diagnosis of kidney disease, diabetes mellitus, heart attack, stroke, and heart failure, and to ascertain whether the person smokes cigarettes or consumes alcohol. In addition, they were trained to ask additional questions about the dietary habits and physical activity of those with elevated blood pressure and record the information on a treatment card.
- *Assess*: The CNs were trained to measure (*assess*) the patients’ weight, height, and waist circumference following standard procedures. In addition, they were trained to initiate medication *treatment* using the simplified Nigerian Hypertension Treatment protocol for primary care, with follow-up every month and in special cases (like non-availability of patients for a while due to travels), bimonthly or quarterly [10].
- *Advise*: CNs were trained on how to *counsel* patients (*advise*) for 20–30 minutes on lifestyle behaviors like incorporating moderate physical activity, weight loss, adherence to clinic visits and medications, and adoption of a healthy diet including increased intake of fruits and vegetables, and reduction of salt intake. Information, education, and communication (IEC) materials were used to aid this aspect of the training.
- *Assist and Arrange*: CNs were trained on how to initiate the *referral* process (*assist*) and to link the patients with elevated blood pressure of 180/110mmHg and above to the appropriate health care facility (*arrange*).

### Training of the PFs using a train-the-trainer model and the 3 E’s

The training of the PFs was based on the train-the-trainer model as they were trained to offer supervisory support to CNs.Training of Trainers (ToT) model is a well-known method of increasing the capacity of the healthcare workforce in LMIC [19]. PFs will be certified and re-certified as trainers yearly by MAP-IT study staff using a train-the-trainer model.

Apart from training on the components of the TASSH protocol, which was held jointly with CNs, the PFs were also trained during the second workshop on the 3 E’s: how to engage the CNs, how to enhance their performance, and how to evaluate their tasks on integrating hypertension treatment into HIV care in their facilities. Specifically, PFs were trained on how to *engage* the CNs via monthly phone calls to address barriers that the CNs may have in performing their duties. PFs were also trained to observe and supervise (*enhance*) the CNs during monthly onsite visits and to *evaluate* the nurses through onsite supervision and the use of online learning communities. The 3E’s were developed specifically for PFs who support nurses delivering TASSH and has been implemented in other TASSH studies [11,20].

### Duration and Mode of Training

Training workshops lasted for roughly six hours and comprised lectures on the prevalence of hypertension in persons living with HIV, how to measure blood pressure, and how to initiate patients on antihypertensive medications using the Simplified Nigerian Hypertension Treatment Protocol. Workshops also included interactive discussions; role plays amongst participants on blood pressure measurements, and initiating TASSH. In addition, there were pre- and post-test evaluations in two of the training workshops, and a comprehensive course evaluation questionnaire was completed confidentially by participants in the first training workshop to assess their view of the training. To further assist the training of healthcare workers, hypertension screening register, hypertension treatment cards, and follow-up appointment cards, developed by the study team, were used for practical demonstrations through role plays and interactive workshops.

### Assessment of the Training

Knowledge acquired by the PFs and CNs was assessed using anonymized pre- and post-training assessment tests completed by participants in two of the three workshops. Assessments included objective questions related to clinical knowledge of hypertension, screening, diagnosis, treatment, and its complications. Additionally, a comprehensive course evaluation questionnaire was completed confidentially by participants in the first workshop to assess participants’ views of the training. They were to score different parts of the training using the Likert scale (4: excellent, 3: very good, 2: good, 1: poor). Descriptive statistics summarized the results of these assessments.

There was no assessment of the third workshop as it was a refresher for the CNs and PFs who attended the first two training workshops and was intended to also create awareness among different stakeholders (e.g., directors of primary healthcare and community health extension workers, CHEWs) on the need to identify and treat hypertension in PLWH.

A paired t-test was used to compare the mean scores pre- and post-test and a p value was used to indicate if the difference was statistically significant or not.

Ethics approval was obtained from the University of Abuja Human Research Ethics Committee and Ibom Multi Specialty Hospital Health Research Ethics Committee.

## Results

### Participants

A total of 92 CNs and six PFs were trained. Eighty-one, 92, and 86 CNs attended the first, second and third training workshops, respectively. A total of six PFs attended each of the three training workshops. Apart from the CNs and PFs, other stakeholders including directors of primary health care, policymakers in Akwa State, CHEWs, pharmacy technicians, and patient support groups attended the workshops as observers and did not participate in the pre-and post-training assessment tests, nor the evaluation of the training workshops.

### Evaluation of knowledge gained and evaluation of the training by participants

Table 1 shows the tools used for evaluation of the workshop. Pre- and post-test scores significantly improved in the first training workshop from a mean score of 11.9 (3.4) to 15.9 (2.9) with p-value < 0.001. Similarly in the second workshop, these test scores improved from 15.4 (1.4) to 16.4 (0.9) with p-value < 0.001 as noted in Table 2. For PFs, pre-and post-test scores also significantly improved in the first workshop from a mean score of 23.6 ± 2.1 to 25.6 ± 1.2, with p-value < 0.01.

**Table 1:** Tools for evaluation of the workshop.


TOOL	DESCRIPTION

Assessment of Knowledge gained by participants	Anonymized pre- and post-training assessment tests completed by participants in two of the three workshops.

	The assessment tests comprised objective questions relating to clinical knowledge of HTN, screening, diagnosis, treatment, and its complications.

	Scores ranged from 0 to a maximum of 20. Each correct response received one point.

Assessment of the training by the participants	A comprehensive course evaluation questionnaire was completed confidentially by participants in workshop one of the training workshops to assess participants’ views of the training.

	They scored different parts of the training using a Likert scale (4: excellent, 3: very good, 2: good, 1: poor).


**Table 2:** Profile of Trainees, Pre-Post Tests Results, and Self-Reported Assessment of Training.


PROFILE	DESCRIPTION	1^st^ COHORT	2^nd^ COHORT	3^rd^ COHORT

Number of trainees	Nurses, *n*	60	86	92

	Practice Facilitators, *n*	06	06	06

Gender distribution of trainees	Female: Male Ratio of Nurses	58/02	84/02	90/02

	Female: Male Ratio of Practice Facilitators	06/0	06/0	06/0

Passive participants	Community Health Extension Workers, Directors of Primary Health Care, Policy Makers, Pharmacy Technicians, Patient Support Group	40	20	86

Knowledge	Description	1st Cohort	2nd Cohort	

Evaluation of Trainees (Nurses and Practice Facilitators	Pre-test, *mean (sd)*	11.9(3.4)	15.4(1.4)	

	Post-test, *mean (sd)*	15.9(2.9)	16.4(0.9)	

	P-value	<0.001	<0.001	

Evaluation of Practice Facilitators	Pre-test, *mean (sd)*	23.6(2.1)	–	

	Post-test, *mean (sd)*	25.6 (1.2)	–	


Table 3 shows the evaluation of the training by participants. Regarding whether the content of the presentation was relevant to the overall objective of the study, 86.1% of the participants reported this as being excellent or very good, while 13.9% felt it was good. The method of training was also evaluated by the participants, with 85.6% feeling that the methodology employed in the overall training was excellent or very good, while the remaining 14.6% felt it was good. Concerning the usefulness of role play in the training workshops, 64.2% of the participants scored the trainings as excellent or very good, while 35.8% scored it as being good. On the impact of the training on improving participants’ knowledge of hypertension, 93.1% of the participants rated it as being excellent or very good, 5.9% rated it as good, while 1.0% rated it as being poor. And on the impact of the training on participants’ overall understanding of the MAP-IT study programme, 86.7% felt that this was either excellent or very good, while 13.3% felt it was good. The level of engagement of participants during the training workshop was also assessed with 81.3% of participants feeling that this aspect of the training was either excellent or very good, 17.8% felt it was good and 0.9% felt it was poor.

**Table 3:** Evaluation of the Training by Nurses, Practice Facilitators, and others *n* = 106.


VARIABLE ASSESSED	DESCRIPTION	LIKERT SCORE, *mean (sd)*	LIKERT 4: n.%	LIKERT 3: n.%	LIKERT 2: n.%	LIKERT 1: n.%

Content Relevance	The content of the presentation(s) was relevant to the overall objective of the meeting (101)	3.25(0.68)	39(38.6)	48(47.5)	14(13.9)	0(0)

Methodology	Usefulness of the training method (104)	3.24(0.69)	40(38.5)	49(47.1)	15(14.4)	0(0)

Role Play	The role-play exercises were quite useful (106)	3.00(0.62)	31(28.7)	64(59.2)	13(12.0)	0(0)

Impact 1	Impact of the training on your overall understanding of hypertension Management (101)	3.29(0.62)	37(36.6)	57(56.4)	6(5.6)	1(0.9)

Impact 2	Impact of the training on your overall understanding of MAP-IT Study (104)	3.17(0.64)	32(30.8)	59(56.7)	14(13.5)	0(0)

Participation	Rating on your concentration, listening, contribution, during the training workshops (106)	3.15(0.74)	37(34.6)	50(46.7)	19(17.8)	1(0.9)


## Discussion

In this manuscript, we describe a tailored training programme for the treatment of hypertension in PLWH for both CNs and PFs in primary healthcare settings in Nigeria. Our training programme for the MAP-IT study is adapted from multiple sources or protocols for hypertension treatment at primary care levels (i.e., WHO PEN package and the Simplified Nigerian Hypertension Protocol for primary care) and well-known methods for delivering behaviour change programmes (i.e., 5As and the Train-the-Trainer model). Previous TASSH studies led by two of the principal investigators for this MAP-IT study have shown the benefits of the training approach for implementing and sustaining TASSH within primary care practices in LMICs [11]. The formation of the MAP-IT training programme from these different sources highlights its significance within the Nigerian context and potentially for other LMIC settings experience increasing prevalence of NCDs among PLWH. Unlike other hypertension training modules for training at the primary healthcare levels in LMICs, which are mainly based on the WHO hearts package, incorporating the simplified Nigeria Hypertension Protocol for primary care made the training more relevant to the local context and thereby may increase the likelihood for TASSH adoption in PHCs for PLWH with uncontrolled hypertension [21].

Additionally, the components of the module which are divided into the ‘5As’ (Ask, Assess, Advise, Assist and Arrange) and mapped onto the identify, counsel, treat and refer (ICTR) approach of managing hypertensive patients living with HIV aided in simplifying the training and adapting it to the local context. The modules and methodology used in our training were acceptable to the trainees as reflected by their evaluation of the programme. Participants positively rated all aspects of the training modules including the impact of the training on improving their knowledge of hypertension and overall understanding of the MAP-IT study programme, and the level of engagement of participants during the training workshop. Although we recognize the limitations of social desirability bias of confidence ratings, with the level of education of these participants, we believe this represents a true reflection of the experiences of CNs and PFs.

Our results showed greater improvement in the knowledge of hypertension in the first training workshop compared to the second training. However, the baseline knowledge of the pre-test score during the second training was higher compared to that during the first training. This can be attributed to the fact that over 80% of the persons attending the second training also attended the first training. Similar improvement between the pre-test and post-test was seen in the study in Mozambique [22].

However, since there was no control group in the pre- and post- analysis, attributing the changes in knowledge alone to the training provided should be viewed with caution.

With a lot of push for countries in Africa to leverage the gains of HIV treatment for NCD management especially the treatment of CVDs, the need for a simplified and contextually adapted training module for this purpose cannot be over-emphasised. Our training programme is an example of such programmes and rightly concentrates on hypertension which is the number one driver for CVDs, and the leading driver for NCDs in most LMICs. For example, Africa, undiagnosed and untreated hypertension remains one of the largest drivers of NCD, and therefore, a roadmap for its prevention and control has been designed [23]. In the 10-point action plan to improve the detection, treatment, and control of hypertension in Africa in the roadmap, the dearth of physician healthcare workers was recognized as a major barrier in reducing the burden of hypertension in Africa and leveraging a task-shifting or task-sharing approach in the management of hypertension was recognized as one of the main ways to overcome this barrier [23].

## Conclusion

Our training methodology showed improvement in the knowledge of managing hypertension in people living with HIV and was highly rated by both CNs and PFs. However, for this approach to be successful there is the need to build capacity for NCD care amongst healthcare providers (including PFs and CNs) workers by incorporating regular trainings into the clinic setting using simplified training modules like ours and testing the effectiveness of these training programmes in both pilot studies and full-scale interventions.
